# Effectiveness of General Medicine in the Management of Rheumatoid Arthritis in Rural Settings: A Systematic Review and Meta-Ethnography

**DOI:** 10.7759/cureus.73726

**Published:** 2024-11-15

**Authors:** Ryuichi Ohta, Chiaki Sano

**Affiliations:** 1 Community Care, Unnan City Hospital, Unnan, JPN; 2 Community Medicine Management, Shimane University Faculty of Medicine, Izumo, JPN

**Keywords:** collaboration, family medicine, general medicine, general practitioners, health services accessibility, primary health care, referral and consultation, rheumatoid arthritis/diagnosis, rural, telemedicine

## Abstract

Rheumatoid arthritis (RA) is a global autoimmune disease causing significant morbidity, particularly in rural areas with limited rheumatology care access. Primary care plays a crucial role in early RA detection and management. This systematic review evaluates RA management in rural primary care to identify ways to enhance quality of care. Following Preferred Reporting Items for Systematic Reviews and Meta-Analyses (PRISMA) guidelines, a meta-ethnography synthesis was conducted on studies from PubMed, Web of Science, and Embase (April 1995-December 2023). Quality was assessed using the best evidence in medical education (BEME) scale, focusing on diagnosis, treatment barriers, and collaboration between general physicians (GPs) and specialists. Of 55 studies, 11 were included. Significant diagnostic delays were linked to patient, physician, and systemic factors. Barriers included limited GP rheumatology training, inefficient referrals, and poor patient-specialist communication. Successful strategies involved GP education, telehealth (e-consults), and collaborative care models. Thematic analysis highlighted patient behaviors, GP knowledge gaps, and the need for interdisciplinary collaboration. Rural RA management faces challenges such as diagnostic delays and limited resources. Key improvements include enhancing GP education, utilizing telehealth, and streamlining referral systems. A multifaceted approach emphasizing the role of primary care in early diagnosis and management is essential to improving RA care in underserved areas.

## Introduction and background

Rheumatoid arthritis (RA) is a chronic autoimmune disorder that significantly impacts the lives of millions worldwide [1]. RA, characterized by painful inflammation and progressive joint damage, poses a complex challenge, especially in rural areas where specialized care is scarce [2]. The global rise in RA cases, combined with difficulties in diagnosis and treatment, underscores the growing burden of this condition [3]. In rural areas, the scarcity of rheumatology specialists places a heavy reliance on general physicians (GPs) as primary caregivers for RA patients [4].

Effective management of RA involves a multidisciplinary approach, including pharmacological treatments, lifestyle modifications, and patient education [5]. However, rural GPs often need more support to navigate these complexities in urban settings [6]. This study aims to fill a critical gap in the literature by focusing on how primary care physicians manage RA in resource-constrained, rural environments. This aspect is crucial as it directly impacts patient outcomes, healthcare resource allocation, and community health [7].

Given the increasing prevalence of RA, particularly in rural contexts, there is a pressing need for more comprehensive research on the role of primary care in managing this chronic disease [8]. This study seeks to synthesize current research and provide evidence-based insights to improve the effectiveness of RA management in rural healthcare systems. Ultimately, the findings aim to inform healthcare policies and improve access to quality care for RA patients in underserved areas.

## Review

This systematic review adhered strictly to the Preferred Reporting Items for Systematic Reviews and Meta-Analyses (PRISMA) guidelines, providing a comprehensive framework for reporting systematic reviews [9]. 

We also performed a qualitative synthesis using the meta-ethnography method to synthesize qualitative data [10,11]. The original articles on meta-ethnography suggest that this method can also synthesize qualitative data in scientific papers. Originally, meta-ethnography was developed to synthesize all qualitative studies that clarified the deep parts of the real world. In clinical medicine, various context-based experiences and specialist knowledge are summarized in the result and discussion part of the original articles. These experiences and wisdom cannot be synthesized using quantitative methods [10,11]. Meta-ethnography can be a valuable methodology to synthesize these data. This process can be applied to qualitatively synthesizing narrative review articles [10,11]. 

A detailed and systematic search strategy was designed to capture relevant literature comprehensively. The search spanned from April 1995 to December 2023, ensuring a broad capture of contemporary research while also including significant historical perspectives. Three major databases were used: PubMed, for its extensive collection of life sciences and biomedical literature; Web of Science, for its multidisciplinary coverage; and Embase, known for its strong focus on drug and pharmaceutical research. These databases are chosen for their breadth, depth, and relevance to rheumatology and primary healthcare.

The search strategy employed a combination of keywords and medical subject headings (MeSH) terms. The keywords included “Rural,” “Remote,” or “Underserved,” combined with “Family Physician,” “General Practitioner,” or “Primary Care,” and further intersected with “Rheumatoid Arthritis” and “Management” or “Treatment.” This approach was designed to be exhaustive and specific, capturing the intersection of RA management in primary care within rural or underserved communities.

Study selection

The inclusion and exclusion criteria are presented in Table 1. 

**Table 1:** Inclusion and exclusion criteria

Criteria	Inclusion	Exclusion
Population	Patients with rheumatoid arthritis (RA), physicians engaged in primary care/family medicine/general practice.	Other people
Setting	Rural or underserved community	Other settings
Types of study	Original articles	Non-empirical studies (editorial, news, review, conference papers)
Interventions	The care and education of rheumatoid arthritis in primary care, family medicine, and general practice	Without primary care, family medicine, and general practice
Outcome	Quality of care of rheumatoid arthritis (RA)	Not health-related
Other	Abstract available, full text available in English	Abstract unavailable, full text unavailable in English

The inclusion and exclusion criteria were meticulously defined to ensure the relevance and quality of the selected studies. Inclusion criteria focused on original articles that provided empirical evidence regarding the management of RA in primary care settings within rural or underserved communities. Exclusion criteria included non-empirical studies such as editorials, news articles, reviews, and conference papers, ensuring the review was based on original research findings.

The process involved an initial screening of titles and abstracts followed by a full-text review of selected articles. Studies were included if they offer insights into the quality of care, patient outcomes, or health service utilization on RA in primary care settings in rural or underserved areas.

Data extraction

Two investigators (RO and CS) conducted a literature search, data extraction, and review to ensure the validity of the review process. Any discrepancies were resolved through discussion. Here, databases were searched for original studies on the care of rheumatoid arthritis in primary care, family medicine, and general practice. Studies without clear descriptions of the aims, participants, or outcomes were excluded (Table 1).

Concretely, the investigators extracted the data from each original study using a purpose-designed data extraction form. The investigators examined the extracted data, which were categorized as follows: country, publication year, participants, purpose, research methodology, health issues, types of outcomes, and outcomes.

Analysis

The quality of each study was assessed based on the Best Evidence in Medical Education (BEME) scale (1 to 5). Grade 1 indicates that no definite conclusions could be drawn, that is, the data are not significant; Grade 2 suggests that the results are ambiguous, but there appears to be a trend; Grade 3 suggests that conclusions could probably be drawn based on the results; Grade 4 indicates that the results are precise and very likely to be accurate; and Grade 5 suggests that the results are unequivocal [12]. The included studies were subjected to a thorough descriptive analysis. This involved summarizing each study in terms of its essential characteristics and findings. The data were compiled into a comprehensive table, providing an overview of the scope and nature of existing research on the topic. This summary table clearly and concisely represents the research landscape, highlighting trends, gaps, and key findings in managing RA in rural primary care settings. To assess the risk of bias in included studies, we used the Newcastle-Ottawa Scale (NOS), which evaluates selection, comparability, and outcome assessment criteria suitable for observational studies. 

Qualitative synthesis respected by meta-ethnography was performed using the following eight steps: getting started, deciding what is relevant to our initial interest, reading the studies, determining how the studies are related, rereading the studies, translating the studies into one another, synthesizing translations, and detailing the synthesis [10,11]. The researcher repeatedly read all the selected articles, extracting the sections relevant to trends, gaps, and key findings in managing RA in rural primary care settings. Vague sections were discussed with the second investigator to decide their inclusion in the analysis. The studies were then translated into one another by inductively coding the extracted content. The researchers thematically synthesized the concepts and themes in each article for translation synthesis. For triangulation, the concepts and themes were discussed among the researchers, and they were also analyzed iteratively during the review period after the completion of a tentative analysis of reviews for theoretical saturation.

Reflexivity

The results of this study were developed collaboratively by the researchers and participants through interactive engagement. The research team comprised individuals with diverse expertise and perspectives on rural medical education. To minimize biases, the research team carefully deliberated on the findings from individual data analyses and considered alternative interpretations while determining the significance of the data.

Selection flow

**Figure 1:**
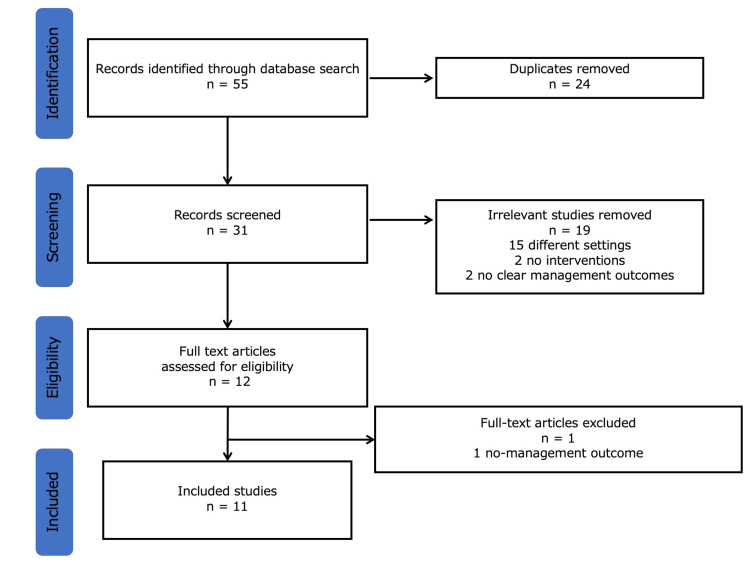
The selection flow

Overall, 55 studies were identified. Of these, 24 duplicate studies were excluded. Upon reviewing the abstracts, we excluded 19 studies due to their different settings (15), lack of interventions (two), and unclear health outcomes (two). Finally, 11 studies were identified in the final analysis after excluding one article through the assessment of eligibility (one, no management outcome) (Figure 1). 

Each article was summarized by year, title, purpose, country, and suggestions regarding primary care and family/general medicine. Details on the articles are presented in Table 2.

**Table 2:** The contents of the included articles DMARDs: disease-modifying anti-rheumatic drugs; USA: The United States of America; RA: rheumatoid arthritis; GP: general physician

Year	Title	Purpose	Country	Suggestions regarding primary care, family/general medicine	Grade
1996 [5]	Management of the early and late presentations of rheumatoid arthritis: a survey of Ontario primary care physicians	Examining primary care physicians' management of rheumatoid arthritis.	Canada	Primary care physicians' investigation of rheumatoid arthritis was in accord with panel recommendations. However, rates of referral to rheumatologists and other healthcare professionals were very low, especially for the early presentation of rheumatoid arthritis.	4
2010 [13]	Optimal care for rheumatoid arthritis: a focus group study	Identify barriers to optimal care for individuals with RA.	Canada	Primary care should be established more comprehensively to take care of patients with rheumatoid arthritis through the education of patients and healthcare professionals.	4
2012 [14]	Use of general practice and rheumatology outpatient services in rheumatoid arthritis	Analyzing the utilization of healthcare among patients with RA.	Estonia	The GP has a significant role in the management of RA patients, especially those who have multiple health problems and those living in rural areas.	5
2012 [15]	Time to consultation and disease-modifying antirheumatic drug treatment of patients with rheumatoid arthritis, northern Alberta perspective	Determining the timeliness of consultation and initiation of DMARD in patients with RA referred to rheumatologists.	USA	Patients with RA are seen and started on DMARD therapy in a timely fashion. Most of the delay is at the primary care level, suggesting a need for improved education of patients and primary care physicians rather than a formal triage system.	5
2013 [16]	Patient's access to healthcare and treatment in rheumatoid arthritis: the views of stakeholders in Portugal	Exploring the views and experiences of Portuguese healthcare stakeholders on key barriers that limit access to treatment and, ultimately, to biologics.	Portugal	Most of the key barriers limiting access to treatment, including biologics, in RA in Portugal are upstream of rheumatology practice. Future actions should focus on primary care education for the management of RA.	5
2015 [6]	A general practice perspective on early rheumatoid arthritis management: a qualitative study from Flanders	Exploring GPs' experiences, beliefs, and attitudes regarding the management of early RA.	Australia	GPs acknowledge the importance of early and intensive treatment but experience various barriers in the management of early RA. GPs should enhance their skills to detect early RA and should actively be involved in early RA care.	5
2016 [17]	From symptoms to diagnosis: an observational study of the journey of rheumatoid arthritis patients in Saudi Arabia	Evaluating the lag times from disease onset to first clinical consultation and diagnosis and identifying factors contributing to delayed diagnosis in Saudi Arabia.	Saudi Arabia	Failure of patients to be seen by rheumatologists at RA onset delayed diagnosis and treatment. Thus, RA diagnosis can be accelerated by encouraging general physicians to learn RA and its management effectively.	5
2019 [4]	Getting a grip on arthritis online: responses of rural/remote primary care providers to a web-based continuing medical education program	An online continuing medical education program to disseminate arthritis clinical practice guidelines was developed to address this issue.	Canada	The online program has demonstrated that it can provide some information rural/remote primary care providers need to deliver optimal care.	4
2021 [18]	Characteristic-specific rates of rheumatoid arthritis and its management in Australian general practice	GP-patient encounters for RA management over the decade, treatments provided for RA management, characteristics of patients managed and of GPs providing management, and differences.	Australia	GP behavior indicates equity and uniformity in RA management nationally. The results suggest adherence to current guidelines for total and new RA contacts.	5
2021 [3]	Journey of rheumatoid arthritis patients in Tunisia: from symptoms to treatment	Assessing the different delays of RA patients' journey from disease onset to treatment initiation and identifying possible influencing factors.	Tunisia	Patients with RA experience a significant delay until diagnosis and initiation of treatment. Primary care providers should urgently provide information about RA to promote effective help-seeking behaviors in patients with arthritis.	5
2023 [19]	Usefulness of an electronic consultation system between primary care health centers and the rheumatology department of a tertiary hospital	Analyzing the demand for rheumatology consultations from primary care and their resolution using the electronic consultation system.	Spain	Thanks to the electronic consultation system, over 40% of queries were resolved in an average of two days; otherwise, patients would have been referred to specialized care.	5

Three studies were assessed as Grade 4, and eight studies were assessed as Grade 5 in terms of the quality of research. We evaluated each study’s potential bias in the selection, performance, detection, attrition, and reporting domains to understand their reliability and applicability, and all articles were reasonable for inclusion in this review.

Demographics of the Included Articles

Of the included articles, three (27.3%) were from Canada; two (18.2%) were from Australia; and one each (9.0%) from Estonia, the United States, Portugal, Saudi Arabia, Tunisia, and Spain. Regarding study design, five studies were cross-sectional (45.5%), three were qualitative (27.3%), one was a cohort study (9.0%), one was interventional (9.0%), and one was a descriptive study (9.0%). Regarding study participants, six studies included RA patients and primary care/family medicine/general practice physicians (54.5%). Other medical professionals, such as rheumatologists, nurses, therapists, and stakeholders in medical institutions, were included in two studies (18.2%). 

Outcome of the included articles

Diagnosis of RA

One study showed that the initial barriers to optimal care for people begin before primary care contact, at the level of the general population, and related to primary care access. The study showed the factors at the patient, physician, and system level influencing how quickly a patient is referred from primary to specialty care. Even after referral, multiple co-management issues influence patient outcomes, such as miscommunication among physicians and other professionals and inadequate resources [13]. Regarding the diagnostic delay, another study showed that the leading causes of this long delay were lack of financial means (33%), wait times until exploration results (31%), wait times until the first GP or rheumatologist visit (26%), and geographical difficulty in accessing healthcare services (18%) [3].

For the initial diagnosis, one study showed that only 3.2% of patients initially sought consultation from a rheumatologist, while 67.2%, 23.6%, and 6.0% first met with orthopedic surgeons, general practitioners, and non-rheumatologists, respectively. Non-rheumatologists offered diagnoses in 24.4% of cases, while rheumatologists diagnosed 75.6%. The absence of early hand/wrist involvement and fatigue were associated with delayed RA diagnosis. Moreover, geographic distribution influenced RA diagnosis, with rural patients experiencing a more significant delay than urban patients [17].

Medical Management of RA

One study shows that the use of GP services was higher among people who lived outside the capital, had more health problems, and experienced disability due to their RA. Time and distance limits affected the frequency of primary and specialist care use. A shorter waiting time to the GP and a longer waiting time to the rheumatologist were associated with more frequent use of GP services [14].

Regarding the barriers to RA management, one study shows that the key barriers identified were the accessibility to primary healthcare services, difficulties in RA diagnosis among GPs, inefficient referral to secondary healthcare, and controlled process of biologic prescription in public hospitals. The leverage points identified included improving epidemiological and clinical knowledge about RA in Portugal, a better understanding of the disease among patients and GPs, clarifying biologic benefits among budget holders, and raising awareness of the current treatment guidelines [16]. 

Regarding statistics of RA management in primary care, proportions of RA management encounters remained static across the decade, were higher for female patients, increased with patient and GP age, increased with socioeconomic disadvantage in major cities, were higher for patients from English-speaking backgrounds, and were higher in regional/remote areas [18].

Collaboration Between Primary Care Physicians and Rheumatologists

Regarding referral, one study shows that primary care physicians would refer suspected RA patients 99.5% of the time, and 87.6% of patients with confirmed RA would have seen a rheumatologist at least once [15]. Another study shows that referrals to physiotherapy, occupational therapy, and rheumatology, all recommended by the panel, were chosen by 206 (38.9%), 72 (13.6%), and 309 (58.4%) physicians, respectively, for early rheumatoid arthritis. For early rheumatoid arthritis, internship or residency training in rheumatology was associated with higher involvement in RA management. On the other hand, for late rheumatoid arthritis, older physicians had higher participation in RA management [5].

One study shows that GPs felt unconfident about their detection skills regarding effective collaboration in RA care. They concretely felt the barriers, such as the limited availability of rheumatology services and long waiting times. Reported key barriers to intensive treatment included patients' resistance and non-adherence, lack of GP involvement, and unsatisfactory collaboration with rheumatology services [6].

Education About RA Management

One study shows the effectiveness of education on RA care for primary care medical professionals. The study shows that participants represented various professions in primary care, including family physicians, physiotherapists, occupational therapists, and nurses, and demonstrated significant improvements in total best practice scores immediately following the completion of the modules [4].

Thematic analysis regarding the effectiveness and challenges in primary/family/general medicine functions in the care of rheumatoid arthritis care

The effectiveness and challenges of primary, family, and general medicine functions in the care of rheumatoid arthritis were analyzed using thematic analysis. Four themes were identified: the patient's difficulty in asking for help, the lack of education and experience in managing RA, the way that these factors affect collaboration between healthcare professionals, and the differences in roles between generalists and specialists (Table 3).

**Table 3:** The result of the thematic analysis RA: rheumatoid arthritis

Theme	Concept
Patient's challenge in help-seeking behaviors delaying patients’ help-seeking behaviors	Lack of recognition of symptoms of RA
	Lack of resources in rural contexts
The lack of education and experience in the management of RA	The differences in experiences of RA
	Lack of training system for rheumatology
Impinging on collaboration among healthcare professionals	The shortfall in referrals to specialists
	Ineffective consultation
Role divisions between generalists and specialists	Collaboration between primary care and rheumatology
	Multimorbidity management from general physicians

**Figure 2:**
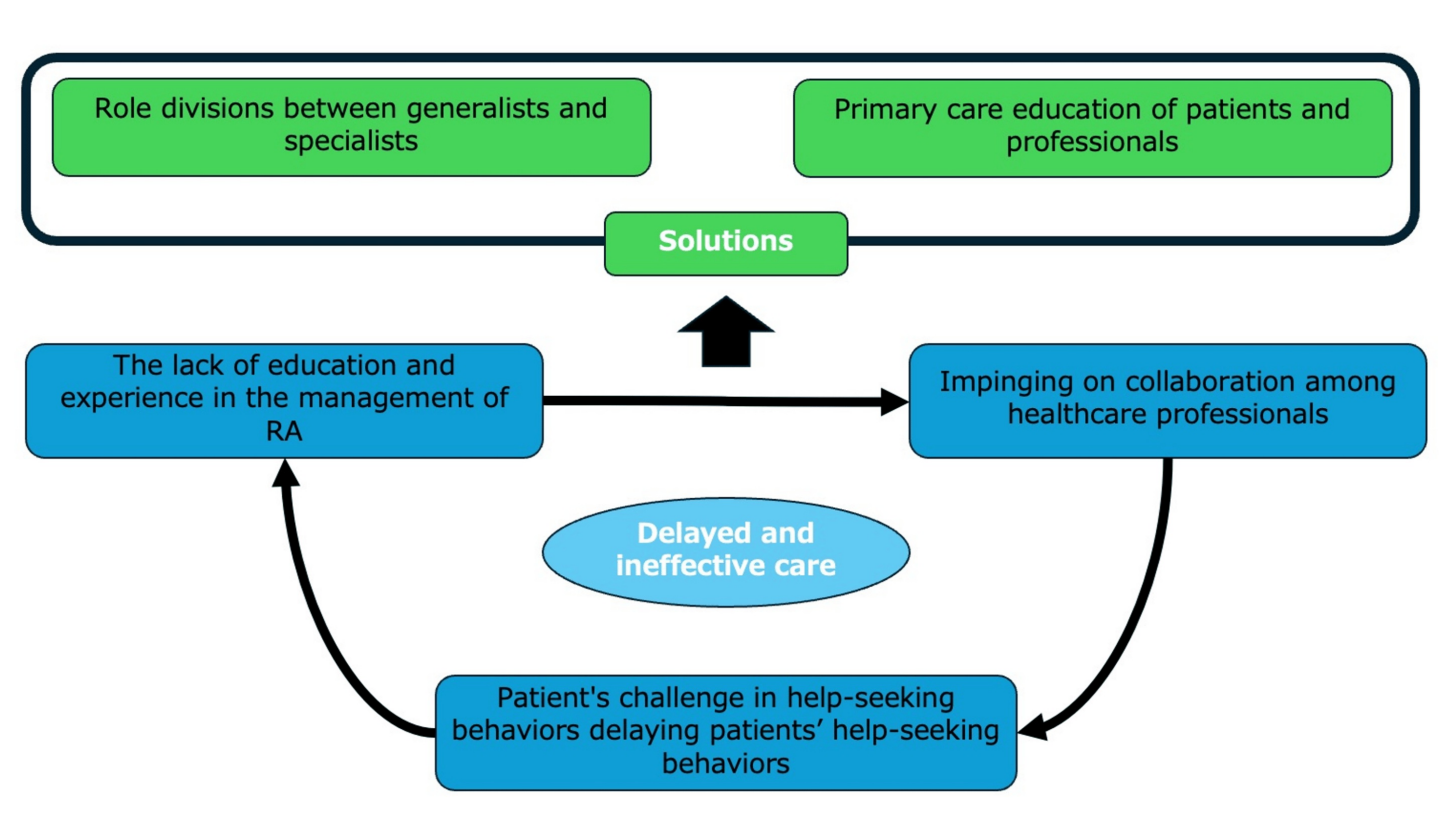
The conceptual figure Image credit: Ryuichi Ohta

The conceptual details of thematic analysis are shown in Figure 2. 

Challenges delaying patients’ help-seeking behaviors

Lack of Recognition of RA Symptoms

As a limitation of early diagnosis and management of RA, ineffective patient help-seeking behaviors (HSB) were suggested. The included article described that patients with RA may not recognize the symptoms of RA efficiently and delay HSB for primary care [12]. As the article explained, regarding barriers before primary care, challenges in RA care begin even before patients contact primary care, rooted in general population awareness and primary care access issues [12]. Another article described that despite the emphasis on early intensive intervention with csDMARDs and "treat-to-target" strategies, most patients experience delays, indicating a gap in achieving optimal disease management in primary care settings because of the delay of patients’ recognition of RA symptoms effectively [3]. As the article shows, enhancing primary care awareness, education, and resources to facilitate earlier RA detection and treatment for rural patients is crucial for preventing disease progression and improving patient outcomes [3]. A fundamental understanding of RA development among rural people is needed to enhance RA management.

The knowledge of RA can be scant among older people in rural contexts. Because of the lack of knowledge, a delay in HSB can occur. As one selected article described, regarding factors influencing delay in diagnosis, patients and family knowledge of RA affected access to care, with rural patients experiencing more significant delays than urban patients, especially among older patients [16]. The lack of patients' and families' knowledge of the progressiveness of RA impinging on their quality of life can defer rural people’s HSB to medical care. The study's results suggested the need for healthcare providers to consider these factors to shorten the RA patient's journey by providing correct information for older patients in rural communities through collaboration with community workers and local governments [16]. 

Lack of Resources in Rural Contexts

Regarding resource optimization, adequate resources are essential for optimizing RA care. The included articles describe various issues related to the lack of resources in rural contexts. One included article described the concrete issues as a lack of resources for education for patients and healthcare providers, communication between all parties involved, and understanding of existing resources [12]. The study underscores the integral role of primary care in initiating and coordinating RA management [12]. It also emphasizes the need for outstanding education and communication, suggesting that primary care can be more effective in RA care with enhanced support and resources [12,13]. For physicians in rural primary care, education about RA for patients and recognition of rural healthcare resources to collaborate with multiple healthcare professionals were considered essential.

Regarding medical resources, three articles described the difficulties in RA diagnosis among GPs: inefficient referral to secondary healthcare, controlled prescription processes in public hospitals, and general accessibility to primary healthcare services. Two articles delineate that the lack of medical institutions may influence patients’ help-seeking behaviors [12,15]. As another article described, factors associated with RA diagnosis delay included rural geographic environment, lack of social insurance, progressive symptom onset, morning stiffness, initial examination by a GP, number of consultations, and number of physicians consulted [3]. Thus, most rural patients tended to perceive the delay in RA diagnosis as long, attributing it also to lack of financial means, wait times for exploration results or physician appointments, and geographic difficulty in accessing healthcare services [3]. Physicians in rural primary care need to recognize the limitations of rural healthcare resources but allocate such resources effectively for detecting and managing RA.

Lack of education and experience in the management of RA

Differences in Experiences of RA

The differences in physicians’ experiences of RA management affected RA patient care in rural primary care and family medicine. The lack of expertise in RA management caused the delay and ineffective management of RA. One of the included articles described primary physicians with specific training in rheumatology as having performed more investigations and interventions for early RA, improving RA management in primary care [5]. Additionally, in the case of late RA, older physicians with more extended experience in RA care showed an affinity and motivation for intervention [5]. Thus, the amount of exposure to RA care among family and primary care physicians affected their self-efficacy of RA care and care quality in rural primary care settings. 

As another included article described, proposed initiatives to address these points include optimizing the RA national registry, disseminating information on rheumatic symptoms in primary care facilities and among the public, increasing interaction between rheumatologists and primary care physicians, broader utilization of disease diagnosis and monitoring tools like DAS28, and implementing hospital-based research to gather real-world data [15]. These approaches can increase the experiences of RA management among medical professionals in primary care settings, improving primary care medical staff’s affinity to RA care. 

Vague symptoms of early RA confused physicians in rural primary settings. Primary and family physicians struggled to approach such patients even though they tried to use variable assessment tools. One of the included articles described how the GPs used multiple assessment techniques for early RA detection and often prescribed non-steroidal anti-inflammatory drugs for suspected cases [6]. However, they expressed a lack of confidence in their detection skills due to unclear symptoms of early RA, inconclusive diagnostic tests, and the low incidence of RA in general practice [6]. For the effective diagnosis of RA in rural primary care settings, physicians needed more experience in RA management in their careers.

Lack of Training System for Rheumatology

Education in rheumatology in primary care settings is scarce, so physicians in primary care settings lack confidence regarding managing RA. There is also a gap between the contents of education for RA in educational institutions and practical demands in primary care. One of the included articles described that the academic gap suggested a potential improvement in primary care, emphasizing the need for enhanced training and awareness about the primary care physician’s role in multidisciplinary care in early RA [5]. The same article described that for primary care physicians in rural contexts, increasing exposure to and knowledge of rheumatology, physiotherapy, occupational therapy, and social work during primary care training is essential for effectively managing RA [5].

Physicians in primary care lack knowledge about epidemiology and clinical expertise and how to update them regarding RA care. One included article described that in overcoming these barriers, there is the need for improved epidemiological and clinical knowledge about RA, a better understanding of the disease among patients and GPs, clarification of biologic benefits among budget holders, and awareness of current treatment guidelines [15]. Rural primary care physicians demand to update their care skills and knowledge to handle RA.

Continual educational interventions about RA management in primary care are thus demanded. The present education regarding RA management in primary care is transient and does not increase the skills and self-efficacy of physicians. As one included study described, while GPs recognize the importance of early and intensive treatment, they face numerous barriers to managing early RA, such as a lack of education and self-efficacy to approach RA [6]. GPs should improve their skills in detecting early RA and become more actively involved in early RA care through comprehensive education on RA [6]. Another study described that while immediate improvements in knowledge were evident through transient educational intervention, the long-term impact and translation of this knowledge into practice still need to be clarified [4]. While the online modules effectively improved immediate expertise regarding best practices in OA and RA care, further research is necessary to determine if these improvements translate into actual changes in clinical practice [4]. This highlights the potential and limitations of web-based educational programs in enhancing the effectiveness of primary/family/general medicine in treating rheumatoid arthritis [4]. The on-site and continual RA education in primary care should be driven for better care of RA patients in rural contexts.

Impinging on collaboration among healthcare professionals

Shortfall in Referrals to Specialists

One of the factors affecting the shortfall in rheumatologist referrals is patient preference. As one of the included articles described, factors such as time and distance impacted primary and specialist care usage frequency [13]. Interestingly, shorter waiting times for GPs and longer distance and waiting times for rheumatologists were linked to more frequent GP visits [13]. In comparison, shorter waiting times for rheumatologists led to more frequent specialist visits [13]. Distance and waiting time for encounters with physicians affected patients’ HSBs in rural contexts. To overcome patients' difficulties, physicians in rural primary care need to accept RA patients smoothly and refer rheumatologists effectively.

Another factor affecting the shortfall is physician factors. One of the included articles described the need for more referrals to specialists and other healthcare professionals, particularly in the early stages of RA [5]. Other research shows that regarding referral rates, primary care physicians referred suspected RA patients 99.5% of the time, with 87.6% of confirmed RA patients having seen a rheumatologist at least once [14]. Primary care physicians’ referral rate was considered low in rural healthcare systems.

Conversely, physicians in rural primary care take into account a variety of referral limitations. One of the included articles described that GPs identified various factors influencing their referral decisions, including limited availability of rheumatology services and long waiting times [6]. Some GPs considered that the initiation of intensive treatment was in the realm of the expertise of rheumatologists [6]. GPs also reported key barriers to intensive therapy, including patients' resistance, non-adherence, lack of GP involvement, and unsatisfactory collaboration with rheumatology services [6]. Another included article described that various factors at the patient, physician, and system levels affected the speed of referral from primary to specialty care, indicating the importance of efficient referral processes in primary care settings for RA management [12]. 

Ineffective Consultation

Timely consultation with rheumatologists can facilitate RA management effectively regarding starting essential drugs for RA, but several factors cause ineffective consultation with rheumatologists. One of the included articles describes that regarding the timeliness of rheumatologist consultation, patients were seen by a rheumatologist an average of 9.8 months after symptom onset and 1.2 months after a referral from a primary care physician [14]. The delay in visiting primary care physicians was caused by ineffective HSB among rural people [14]. The patient's ineffective HSB delayed the detection of RA, eventually causing delayed consultation from primary care to rheumatologists.

Promptly starting medication for RA is critical for the prevention of the progression of RA. Smooth treatment can be possible when effective consultation is possible [12,14]. Regarding the initiation of DMARD therapy, as one of the included articles shows, all referred patients with confirmed RA started DMARDs within one week of the initial rheumatologist consultation, which can contribute to the effective management of RA [14]. However, the negative image of DMARDs among physicians and patients in rural primary care affected the preference for DMARDs in primary care. DMARDs are used for the treatment of cancers and other critical diseases, so primary care physicians tended to show negative attitudes toward the medicines, and such attitudes affected the HSBs of rural patients [12]. The delay in the HSBs of patients and the negative attitudes of rural physicians toward DMARD treatments were limitations [12,14]. Effective consultation of primary care physicians for rheumatologists in rural contexts was demanded based on better HSB and an appropriate understanding of RA treatments [12,14].

Role divisions between generalists and specialists

Collaboration Between Primary Care and Rheumatology

There is an effective collaboration between primary care physicians and rheumatologists for the effective care of RA in rural primary care. Post-referral management is especially essential for the effective control of RA activities. As one of the articles described in the included articles, in post-referral co-management, numerous co-management issues arise after patients are referred to specialists, influencing patient outcomes [12]. Another article described this issue and highlighted the need for effective coordination between primary care and specialists in managing RA through effective communication and role division between primary care and rheumatology [12,13].

For effective collaboration, physicians in rural primary care need a primary role in early RA management, especially in early diagnosis, referral, and post-referral management. One of the included articles describes that regarding the effectiveness of primary care in RA care, a substantial gap in the early diagnosis, referral to rheumatologists, and post-referral management of RA by GPs should be overcome [16]. Primary care physicians, including GPs, play a limited role, especially in the post-referral management of RA regarding the continuity of medications and complication management of RA [16]. Another article described that physicians in rural primary care should take the role of post-referral management of RA, and the post-referral management of RA should be driven because rural citizens may not frequently go to rheumatology clinics owing to their socioeconomic conditions [12,13].

For effective post-referral management, e-consults were suggested by several articles on the effective collaboration between primary care physicians and rheumatologists. One of the included articles described that e-consultation is expanding because of the COVID-19 pandemic; patients were used to e-consult, so the effectiveness of e-consults in primary care for managing rheumatic diseases, particularly in RA, was clarified worldwide [18]. The quick response time and high-resolution rate of queries highlighted the potential of e-consults in improving patient care and reducing the burden on specialized services of RA [18]. Thus, as some articles described, strengthening the capability of primary care practitioners to recognize early symptoms of RA and improving their referral practices to specialists using various communication tools can contribute to better care of RA in rural contexts [16,18]. Increasing awareness among GPs about the critical need for early intervention in RA and establishing better coordination between primary care and rheumatology services are mandatory in rural RA care [16].

Multimorbidity Management From General Physicians

The strong points of primary care and general medicine are emphasized regarding multimorbidity. Multimorbidity is one of the critical factors for controlling older patients’ health conditions [20]. General physicians can effectively control older people’s multimorbidity. Older patients with RA and multimorbidity in rural contexts should be taken care of, mainly by general physicians. One of the included articles describes that the use of GP services is higher among those living outside the capital, with more health problems and experiencing disability due to RA [13]. The same article highlights the significant role of GPs in managing RA, especially for patients with multiple health issues and those in rural areas. Another article describes that primary care functions effectively in RA care, adapting to patient needs based on location, health status, and accessibility factors [17].

In primary care settings, there is an increased population of older RA patients cared for by primary care providers because of their multimorbidity, so primary care physicians continually need to develop the comprehensive management skills of RA. One of the included articles describes that the proportion of RA management encounters remained constant throughout the decade and gradually increased among the older population. Still, there was an increase in RA management encounters with the aging of patients and GPs [17]. Another article describes GP behavior, indicating nationwide equity and uniformity in RA management. GPs adhere to current guidelines for total and new RA patient contacts for the quality of RA care among older patients [14]. The education of patients and primary care physicians respecting older patients’ issues, such as multimorbidity and polypharmacy, needs to be improved for early detection, referral, and post-referral management [14,17].

Discussion

This systematic review and meta-ethnography offer essential insights into RA management in rural primary care by identifying actionable strategies to address well-known challenges. Unlike previous studies that broadly call for better communication and earlier diagnosis, our research highlights rural healthcare providers' specific barriers, such as limited access to rheumatology specialists and the need for targeted education for GPs. Innovative solutions like e-consults are emphasized to bridge gaps in specialist care and community-based educational programs to enhance patient awareness. Our findings provide a detailed roadmap for improving RA management in these underserved areas.

Diagnostic delays in RA have significant consequences, including irreversible joint damage, increased disease activity, and reduced functional capacity [20,21,22]. Our findings align with existing evidence, stressing the importance of early diagnosis within the first few months of symptom onset to improve outcomes.

Barriers contributing to delays include patient unawareness of RA symptoms, often attributed to aging or physical strain, leading to delayed help-seeking [23]. Socioeconomic factors, such as financial constraints, exacerbate this issue, causing patients to prioritize other concerns [7]. For GPs, the nonspecific presentation of RA and time constraints make early diagnosis difficult [24,25]. The rarity of RA in comparison to other conditions, compounded by healthcare system factors like long wait times for specialist referrals, further impedes timely diagnosis and treatment initiation [6,14,26,27].

Increasing patient awareness through public health campaigns and community health programs is critical to encouraging earlier consultation [22,28]. Physician education and training in rural settings should be enhanced, equipping GPs with tools for early RA detection and timely referral [29]. Continuing medical education and decision-support systems can improve GP confidence in managing suspected RA cases.

Streamlining referral pathways and expanding access to rheumatology services are crucial for addressing diagnostic delays. Centralized referral systems, fast-track clinics for early arthritis, and telemedicine consultations are recommended strategies to reduce delays [30]. These approaches ensure that RA patients in rural areas receive prompt specialist care.

Primary care is the first point of contact in managing RA, with early detection and referral being critical for patient outcomes. However, effective RA management in primary care depends on GP knowledge, referral efficiency, and access to rheumatology services [4,6,31]. The lack of GP training in RA, particularly in its early stages leads to diagnostic delays. GPs may struggle to distinguish RA from other musculoskeletal conditions, leading to referral delays [13,31]. The referral process can be a bottleneck, with long wait times for rheumatology appointments delaying treatment [6,16].

Enhancing GP education on RA management is essential. Continuing medical education programs and online resources, such as the European League Against Rheumatism (EULAR) courses, can improve GP knowledge and confidence [6,16,32]. Referral systems should be streamlined to allow direct access to suspected RA cases, enabling faster specialist referrals without bureaucratic hurdles [3,8]. Integrating electronic health records (EHRs) with decision support tools can help GPs identify and refer RA cases more efficiently [16].

Telemedicine offers a solution to geographic barriers in rural settings, enabling virtual consultations that expedite specialist input. This reduces the need for face-to-face consultations, facilitating earlier intervention [4]. E-consults have improved communication between GPs and specialists, allowing for more rapid decision-making and treatment adjustments [4,33]. E-consults reduce patient wait times and provide GPs with ongoing support, enhancing their competence in managing RA.

Interdisciplinary collaboration between GPs, rheumatologists, and allied health professionals, such as nurses and physiotherapists, is also essential. This collaborative approach ensures comprehensive care for RA patients, addressing medical and psychosocial needs [29,34,35].

Public awareness campaigns are crucial for educating patients about RA symptoms and encouraging earlier consultation. Continuing education for GPs can improve their ability to recognize RA symptoms, understand the disease's progression, and refer patients promptly [36-38]. E-consults offer an effective tool for facilitating specialist input, especially in managing complex cases or multimorbidity [39-41]. Fast-track referral systems can also mitigate delays, ensuring patients receive timely care [42].

This study has several limitations. First, reliance on published literature may introduce publication bias, as studies with positive results are more likely to be published, affecting the comprehensiveness of our findings. Second, the focus on articles available in major databases may overlook relevant studies from lesser-known journals or languages other than English. Lastly, the qualitative synthesis relies on subjective interpretation of the literature, which can introduce bias. Additionally, the rapidly evolving nature of RA management and telehealth may limit the long-term applicability of the findings. Despite these limitations, this study provides valuable insights into improving RA care in rural settings. The presence of selection bias in some studies may influence the representativeness of our findings, particularly in rural healthcare settings. Future studies should prioritize minimizing bias to improve data reliability.

## Conclusions

This systematic review and meta-ethnography identifies key challenges and opportunities in RA care within primary/family/general medicine settings. Diagnostic delays, driven by patient unawareness, limited GP education, and healthcare system barriers, remain significant issues. Additionally, the prevalence of multimorbidity complicates RA management, necessitating the need for comprehensive care approaches. The study highlights the potential of digital health technologies like e-consults to streamline referrals and improve collaboration between GPs and rheumatologists, especially in the post-COVID-19 era. Education and training are crucial for equipping healthcare providers with the skills needed for early diagnosis and effective RA management. Actionable strategies include increasing public and professional awareness, optimizing referral pathways, and leveraging telehealth solutions to enhance RA care. Future research should evaluate the long-term impact of these interventions and explore how socio-economic and geographic factors affect access to care. By focusing on education, collaboration, and technology, healthcare systems can improve outcomes for RA patients.
